# Sensitivity and specificity of blood-fluid levels for oral anticoagulant-associated intracerebral haemorrhage

**DOI:** 10.1038/s41598-020-72504-7

**Published:** 2020-09-23

**Authors:** Abeer Almarzouki, Duncan Wilson, Gareth Ambler, Clare Shakeshaft, Hannah Cohen, Tarek Yousry, Rustam Al-Shahi Salman, Gregory Y. H. Lip, Henry Houlden, Martin M. Brown, Keith W. Muir, Hans Rolf Jäger, David J. Werring

**Affiliations:** 1 Physiology Department, Faculty of Medicine, King Abdulaziz University, Jeddah, Saudi Arabia; 2 Department of Brain Repair and Rehabilitation, UCL Stroke Research Centre, UCL Institute of Neurology and the National Hospital for Neurology and Neurosurgery, Russell Square House, 10-12 Russell Square, London, WC1B 5EH UK; 3 Department of Statistical Science, University College London, Gower Street, London, UK; 4 Haemostasis Research Unit, Department of Haematology, University College London, 51 Chenies Mews, London, UK; 5 Lysholm Department of Neuroradiology and the Neuroradiological Academic Unit, Department of Brain Repair and Rehabilitation, UCL Institute of Neurology, Queen Square, London, UK; 6 Centre for Clinical Brain Sciences, School of Clinical Sciences, University of Edinburgh, Edinburgh, UK; 7 Liverpool Centre for Cardiovascular Science, University of Liverpool and Liverpool Heart and Chest Hospital, Liverpool, UK; 8 Aalborg Thrombosis Research Unit, Department of Clinical Medicine, Aalborg University, Aalborg, Denmark; 9 Institute of Neuroscience and Psychology, University of Glasgow, Queen Elizabeth University Hospital, Glasgow, UK; 10 Department of Molecular Neuroscience, UCL Institute of Neurology and the National Hospital for Neurology and Neurosurgery, Queen Square, London, WC1N 3BG UK

**Keywords:** Cerebrovascular disorders, Stroke

## Abstract

Intracerebral haemorrhage (ICH) is a life-threatening emergency, the incidence of which has increased in part due to an increase in the use of oral anticoagulants. A blood-fluid level within the haematoma, as revealed by computed tomography (CT), has been suggested as a marker for oral anticoagulant-associated ICH (OAC-ICH), but the diagnostic specificity and prognostic value of this finding remains unclear. In 855 patients with CT-confirmed acute ICH scanned within 48 h of symptom onset, we investigated the sensitivity and specificity of the presence of a CT-defined blood-fluid level (rated blinded to anticoagulant status) for identifying concomitant anticoagulant use. We also investigated the association of the presence of a blood-fluid level with six-month case fatality. Eighteen patients (2.1%) had a blood-fluid level identified on CT; of those with a blood-fluid level, 15 (83.3%) were taking anticoagulants. The specificity of blood-fluid level for OAC-ICH was 99.4%; the sensitivity was 4.2%. We could not detect an association between the presence of a blood-fluid level and an increased risk of death at six months (OR = 1.21, 95% CI 0.28–3.88, *p* = 0.769). The presence of a blood-fluid level should alert clinicians to the possibility of OAC-ICH, but absence of a blood-fluid level is not useful in excluding OAC-ICH.

## Introduction

Oral anticoagulant-associated intracerebral hemorrhage (OAC-ICH) is a devastating disease^1^, with a reported 90-day case fatality of 42%^2–4^. The incidence of OAC-ICH is growing substantially, with increased use of anticoagulant therapy^5^. 5–12% of ICH is related to OAC^6^, and is expected to increase with an ageing population increasingly exposed to oral anticoagulants^7^. The risk of haematoma growth after OAC-ICH is as high as 54%^8^. Rapid identification of patients with OAC-ICH is important to allow rapid coagulation reversal, strict blood pressure management and transfer to a higher dependency unit^9^.

The identification of patients with OAC-ICH can be challenging, for example, when patients cannot communicate and there is no clear history from an informant. Computerized tomography (CT) remains the initial neuroimaging tool of choice for identification of acute ICH^10^. The presence of a blood-fluid level has been suggested as a marker for OAC-ICH^11,12^, but with the exception of one study (a sub-study of INTERACT-2) most published studies are case reports or were done on small samples^11–15^. In a study of 2065 patients from the INTERACT-2 study, blood-fluid levels on baseline CT (found in 19 patients in the sample) were associated with the use of warfarin as well as poor outcome 90 days after ICH^16^.

In this study, we aimed to determine the prevalence, sensitivity and specificity of blood-fluid levels as a marker for OAC-ICH and its prognostic significance in a large multicentre prospective cohort of patients with ICH.

## Methods

### Participants

We included participants with CT-confirmed acute ICH (scanned within 48 h of symptom onset) recruited in the observational Clinical Relevance of Microbleeds in Stroke Study (CROMIS-2) conducted at 79 hospitals throughout the UK (and one in the Netherlands) between 2012 and 2015. The protocol has been published elsewhere^17^. Briefly, patients were eligible if they were 18 years or older and had a spontaneous ICH not secondary to major trauma or a macrovascular cause, as previously described^18^.

The CROMIS-2 study was approved by the UK National Health Service Research Ethics Committee and was carried out in accordance with the Declaration of Helsinki. Patients with capacity gave informed written consent. When patients could not consent, it was obtained in written form from a proxy (as defined by relevant local legislation).

### Imaging

**Figure 1:**
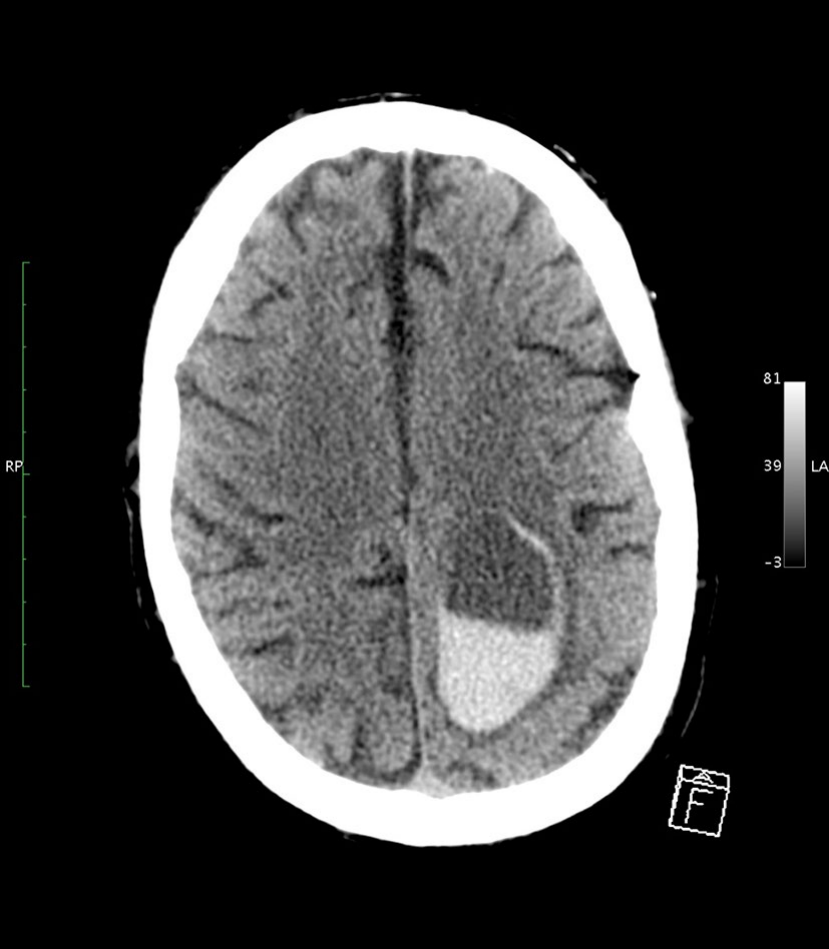
Typical blood-fluid level on an acute CT scan of a patient with an intracerebral hemorrhage in the left parietal lobe.

CT imaging was performed within 48 h of ICH onset in all patients. Digital CT images were collected in uncompressed Digital Imaging and Communication in Medicine (DICOM) format and analysed centrally. CT scans were examined for the presence of a blood-fluid level by two researchers (blind to anticoagulant status and clinical outcome). The raters were a neuroscience graduate student (A.A.) and a vascular neurologist (D.W.) with experience in neuroimaging research—A.A. performed the initial ratings, which were then checked and discussed with D.W.. As described previously^11^, a blood-fluid level was defined according to the following features: (1) upper compartment hypodense to the brain, (2) lower compartment hyperdense to the brain and (3) a sharply defined horizontal interface between the upper and lower compartments (Fig. 1). ICH location was classified as infratentorial, deep, or lobar (cortical or cortical–subcortical) using a validated rating instrument^19^.

### Baseline and follow up data

Data collected at baseline included age, sex, ethnicity, pre-morbid function assessed by the modified Rankin scale, clinical information, history of comorbidities (e.g., hypertension, atrial fibrillation), history of previous ischaemic stroke, ICH or TIA), and use of antithrombotic drugs prior to ICH. We recorded examination findings on admission, systolic and diastolic blood pressure, Glasgow coma scale score, the international normalized ratio (INR), and the use of surgical intervention. Mortality was assessed at six months post-ICH using information provided by the National Health Service digital data (Health and Social Care Information Centre)^17^.

### Statistical analyses

We performed statistical analyses using SPSS version 24 and R version 3.4.3. We visually inspected the distribution of the data using histograms for continuous variables. We analysed data that were not normally distributed using appropriate non-parametric tests. The results of this study were expressed as median and interquartile range (IQR) for continuous variables and as numbers and percentages (%) for categorical variables. We divided the sample into a blood-fluid level group and a non-blood-fluid level group. We tested the differences between the two groups using independent-sample Mann–Whitney U tests for continuous variables, while Fisher’s exact test was used for categorical variables. The assumptions of these tests were checked and the corresponding effect sizes are reported. The effect sizes used were r for the Mann–Whitney U test (the Z-value divided by the square root of the total number of observations)^20^ and the odds ratio for the chi-squared test.

We calculated the sensitivity, specificity, and positive and negative predictive values of fluid level for anticoagulation (as well as their 95% confidence intervals; 95% CI). We tested the association between the anticoagulant status and CT-defined blood-fluid level using a chi-squared test. The association between the presence of a blood-fluid level and various clinical and imaging parameters was assessed using logistic regression. The association between blood-fluid level and six-month mortality was assessed using logistic regression with adjustment for prespecified clinically important variables known to be associated with outcomes: age, sex, premorbid mRS, ICH location, hematoma volume, intraventricular extension of the hemorrhage, and oral anticoagulant use. All model assumptions were checked. For all analyses, *p* < 0.05 (two-tailed) was considered significant.

## Results

**Figure 2:**
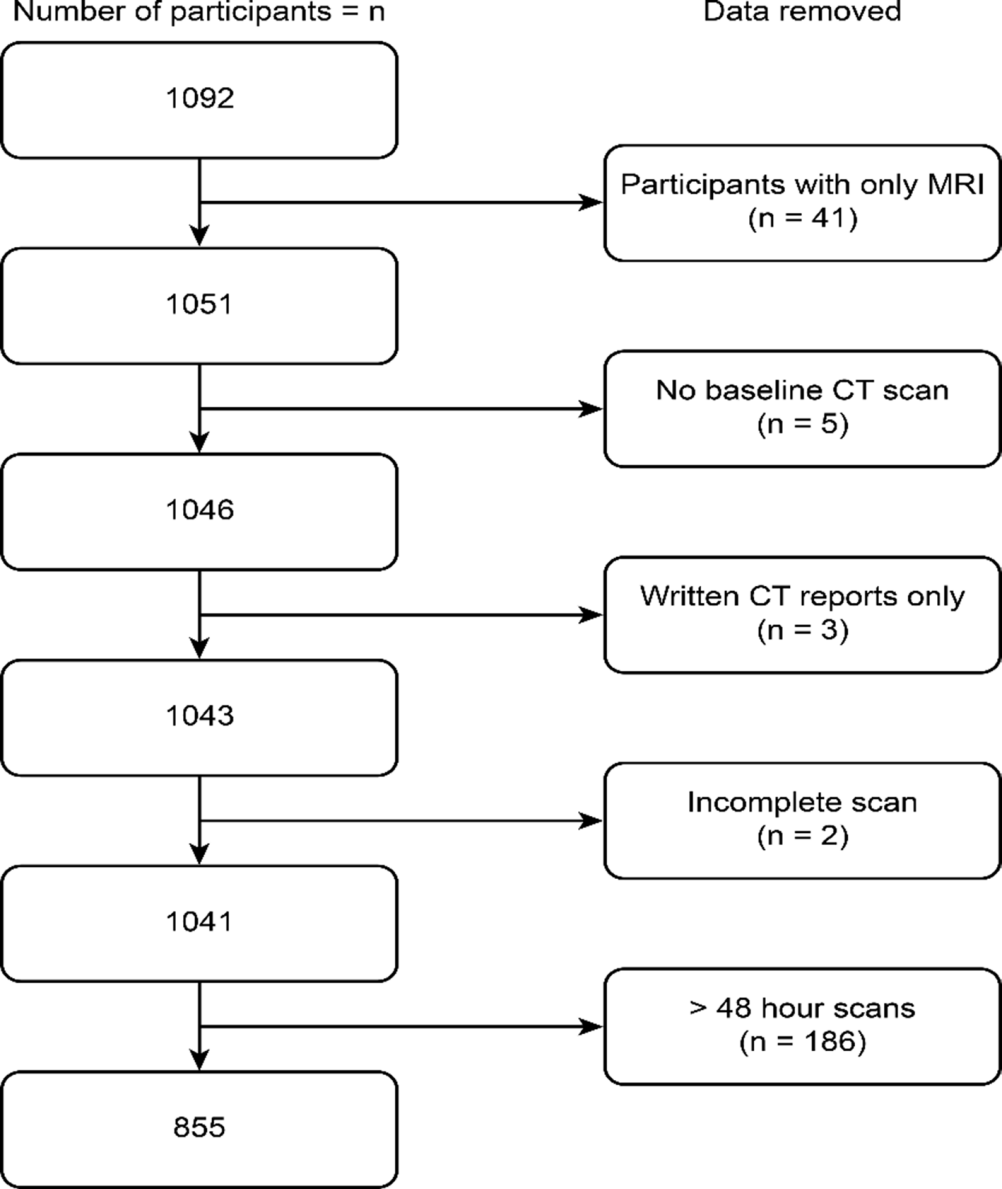
Flowchart showing the recruitment of patients for this study, including reasons for exclusion.

We included 857 patients with a median age of 75.5 years (IQR = 65.8–82.4), of whom 361 (42.1%) were female (Fig. 2). Anticoagulant status was unknown in six patients in the study sample, none of whom had a blood-fluid level. Of the 360 patients on anticoagulant therapy in the study sample, 20 were on a Factor Xa inhibitor, two were on Dabigatran, and the rest were on warfarin.

A blood-fluid level was demonstrated in eighteen patients (2.1%) by acute CT imaging (within 48 h of the ICH). Table 1 summarises the characteristics of the participants between the two groups. Compared to the non-blood-fluid level group, the blood-fluid level group had a significantly higher INR (3.05 (2.23–3.9) versus 1.10 (1.00–2.30), *p* = 0.0001, r = 0.13). The blood-fluid level group were more often taking an anticoagulant (warfarin in all cases) at ICH onset than the non-blood-fluid level group (15 [83.3%] versus 345 [41%]; *p* = 0.0004). The blood-fluid level group also underwent OAC reversal more frequently, in 13 (72.2%) versus 256 (30.5%) (*p* = 0.0005) and had significantly more atrial fibrillation (14 [77.8%] compared to 289 [34.4%], *p* = 0.0008) in the non-blood-fluid level group. Overall, there was no statistically significant difference in death at six months post-ICH between the blood-fluid level group and the non-blood-fluid level group (3 [20.0%] vs. 124 [14.8%], *p* = 0.7312).

**Table 1:** Patient baseline and follow up characteristics in each group.

Characteristic	Total number n = 857	Fluid level n = 18	Non fluid level n = 839	*p* value
Age (years) (median, IQR)	75.5 (65.8–82.4)	79.5 (70.5–83.0)	75.5 (65.6–82.3)	0.2588
Male n, (%)*	496 (57.9%)	14 (77.8%)	482 (57.4%)	0.0957
White background n, (%)	743 (86.7%)	15 (88.2%)	728 (86.8%)	0.5657
**Anticoagulants n, (%)**	**360 (42.0%)**	**15 (83.3%)**	**345 (41.1%)**	**0.0004**
*Comorbidities*				
**Atrial fibrillation n, (%)**	**303 (35.4%)**	**14 (77.8%)**	**289 (34.4%)**	**0.0008**
Hypertension n, (%)	566 (66.0%)	12 (66.7%)	554 (66.0%)	1.0000
*History of previous stroke*				
IS n, (%)	118 (13.8%)	4 (25.0%)	114 (13.6%)	0.2647
ICH n, (%)	35 (4.1%)	2 (12.5%)	33 (3.9%)	0.1430
TIA n, (%)	98 (11.4%)	1 (6.7%)	97 (11.6%)	1.000
*Medication on admission*				
Aspirin n, (%)	189 (22.1%)	1 (5.9%)	188 (22.4%)	0.1403
**Warfarin n, (%)**	**323 (37.7%)**	**14 (77.8%)**	**309 (36.8%)**	**0.0008**
*Clinical measurements*				
SBP (median, IQR)	166 (147–190)	160 (140–172)	167 (147–190)	0.1475
DBP (median, IQR)	90 (79–105)	83 (78.5–91.5)	90 (79–105)	0.2745
GCS (median, IQR)	15 (14–15)	15 (14–15)	15 (14–15)	0.8221
*Blood results on admission*				
**INR (median, IQR)**	**1.11 (1.00–2.40)**	**3.05 (2.23–3.90)**	**1.10 (1.00–2.30)**	**0.0001**
*Management received*				
Surgery n, (%)	29 (3.4%)	2 (11.1%)	27 (3.2%)	0.1242
**AC reversal n, (%)**	**269 (31.4%)**	**13 (72.2%)**	**256 (31.1%)**	**0.0005**
Haematoma location**				0.0524
Lobar n, (%)	296 (34.5%)	10 (55.6%)	286 (34.1%)	
Deep n, (%)	459 (53.6%)	5 (27.8%)	454 (54.1%)	
Infratentorial n, (%)	79 (9.2%)	3 (16.7%)	76 (9.1%)	
**Haematoma volume (median, IQR)**	**7.1 (2.4–17.5)**	**14.1 (5.5–23.7)**	**7.0 (2.3–17.2)**	**0.0451**
Intraventricular extension n, (%)	249 (29.1%)	2 (11.1%)	247 (29.4%)	0.1345
Deceased n, (%)	127 (14.8%)	3 (20.0%)	124 (14.8%)	0.7312

*ICH* intracerebral haemorrhage, *TIA* transient ischemic attack, *SBP* systolic blood pressure, *DBP* diastolic blood pressure, *GCS* Glasgow coma scale, *INR* International normalized ratio, *AC* anticoagulant.

*Note that data on sex was missing from one patient in the sample.

**Note that data on hematoma location was missing from 23 patients in the sample. The differences between the groups were statistically significant (*p* < 0.05) for the variables shown in bold. IS: ischemic stroke.

### Blood-fluid level sensitivity and specificity to OAC-ICH

Of the patients in whom a blood-fluid level was seen (n = 18), the majority (15/18 (83.3%) were on anticoagulants (see Supplementary Table 1). The non-blood-fluid level group consisted of 839 patients. Less than half of this group was on anticoagulants (345/839, 41%). The presence of a blood-fluid level was significantly associated with the use of anticoagulants (χ^2^ = 12.6, *p* < 0.001, odds ratio [OR] 7.1, 95% CI 2.0–38.6; *p* < 0.001).

The presence of a blood-fluid level identifies just 4% (95% CI 2–7%) of patients on anticoagulants (sensitivity). However, the absence of a blood-fluid level identifies 99% (95% CI 98–100%) of patients who are not on anticoagulants (specificity). Of all the patients with a blood-fluid level, 83% were on anticoagulants (positive predictive value, 95% CI 59–96%). The negative predictive value, the probability that patients with no blood-fluid level were not on anticoagulants, was 59% (95% CI 55–62%).

### Haematoma characteristics of OAC-ICH associated with blood-fluid level on CT

Haematomas of the patients in the blood-fluid level group (median = 14.1, IQR = 5.5–23.7) were significantly larger than those of patients in the non-blood-fluid level group (median = 7.0, IQR = 2.3–17.2) (*p* = 0.045). Blood-fluid level-associated haematomas tended to have a lobar location (55.6%), but there were no statistically significant differences in haematoma location between the groups (*p* = 0.0524).

### Factors associated with blood-fluid level in patients on anticoagulants

In a multivariate logistic regression including age (OR 1.00, 95% CI 0.95–1.97, *p* = 0.867), sex (OR 2.68, 95% CI 0.70–13.38 , *p* = 0.176), haematoma location (OR 0.93 , 95% CI 0.391–2.04 , *p* = 0.858), haematoma volume (OR 0.99, 95% CI 0.96–1.02, *p* = 0.775), intraventricular extension (OR 0.15, 95% CI 0.007–0.93, *p* = 0.097), hypertension (OR 1.05 , 95% CI 0.31–4.34, *p* = 0.944), and platelet count (OR 1.00, 95% CI 0.99–1.01, *p* = 0.559), only the INR was significantly associated with the presence of a blood-fluid level in patients on anticoagulants (OR 1.58 , 95% CI 1.08–2.34 , *p* = 0.0174).

### Prognostic significance of blood-fluid level on mortality

Six hundred and ninety-nine patients (81.6%) had follow up at 6 months and complete data for the logistic regression analysis. In this analysis, the presence of a blood-fluid level was not significantly associated with higher odds of death at six months (OR 1.21, 95% CI 0.28–3.88, *p* = 0.7690). In a multivariable analysis, older age, higher pre-morbid mRS, larger haematoma volume, intraventricular extension, and oral anticoagulant use were all associated with higher mortality (Table 2).

**Table 2:** Association between blood-fluid level and mortality.

	OR	95% CI	*p* value
*Unadjusted model*			
Blood-fluid level (yes)	1.21	0.28–3.88	0.7690
*Fully adjusted model*			
Blood-fluid level (yes)	0.82	0.67–3.08	0.7852
**Age**	**1.06**	**1.03–1.08**	** < 0.0001**
Sex (female)	0.97	0.61–1.54	0.8903
Location (lobar)	0.99	0.66–1.47	0.9549
**Pre-morbid MRS**	**1.52**	**1.28–1.81**	** < 0.0001**
**Hematoma volume**	**1.02**	**1.01–1.03**	** < 0.0001**
**Intraventricular extension**	**2.13**	**1.32–3.43**	**0.0018**
**Oral anticoagulant use (yes)**	**1.84**	**1.18–2.91**	**0.0080**

Reference categories are shown in parentheses for the categorical variables in the models.

## Discussion

In this study, we found a blood-fluid level in 18 out of 855 (2%) patients with acute ICH using CT imaging. A blood-fluid level was associated with the use of anticoagulants with a high specificity (99%) but low sensitivity (4%). Patients with a blood-fluid level also had larger ICH volumes but no evidence of higher mortality.

Our reported prevalence of a blood-fluid level is similar to that in a recent large ICH study that reported a prevalence rate of 1%^16^; however, no sensitivity level was reported in their study. In terms of diagnostic agreement, our results differed somewhat from those reported in previous studies. The sensitivity of the blood-fluid level for coagulopathy was found to be 59.4% in the study by Pfleger et al.^11^ and 41.6% in the study by Gökce et al.^12^, while it was considerably lower (4%) in our study. However, Pfleger et al.’s study did not group patients based on use of OACs, but based on the presence of coagulopathy in general. Pfleger et al.’s study included patients on warfarin, patients with liver failure, diffuse intravascular coagulation, and other causes of coagulopathy, the pathophysiology of which may be different to OAC-associated ICH. In addition, our study had a substantially larger sample size than previous studies and included patients on non-vitamin K antagonist oral anticoagulants, which may have influenced the sensitivity estimates.

Although there have been a few case reports of a blood-fluid level in patients with no underlying coagulopathy^21–23^, our findings suggest that in acute ICH a blood-fluid level is highly specific for OAC-ICH (99%), consistent with previous clinical studies^11,12,16^. We have also confirmed previous observations that a blood-fluid level is associated with larger haematoma volume^11,12,16^, which is associated with worse clinical outcome^24^. The presence of a blood-fluid level was not associated with mortality at six months in our study. This result suggests that the value of this sign is in diagnosing intracerebral hemorrhage associated with anticoagulants, but that it has no prognostic significance. This is at odds with the results from the INTERACT-2 data^16^, however, the relatively low prevalence of patients with blood-fluid level in both these studies may have led to spurious results. As previously suggested^16,25^, a possible association between blood-fluid levels and increased mortality may be related to faster haematoma growth, but this needs to be further investigated in larger, longitudinal imaging studies.

Our study has strengths. We recruited a large cohort of ICH patients from 80 hospitals, so our findings should be widely generalisable. We used a strict definition of blood-fluid level, included only acute (< 2 days) CT scans, and performed the blood-fluid level rating blinded to anticoagulant use and outcome. The main limitation of our study is the small number of participants in the blood-fluid level group, which limits its statistical power. Although our study suggests that higher INR is associated with a higher prevalence of blood-fluid level in patients on anticoagulants, larger future cohorts are needed to investigate the relationship between INR and blood-fluid level in more detail, to determine whether the presence of a blood-fluid level occurs mainly with INRs outside the therapeutic range. One mechanism proposed to explain this is that, in the absence of normal blood clotting, red blood cells fall to the bottom of the haematoma, leaving plasma at the top, causing a sedimentation level. If this mechanism underlies blood-fluid levels, clotting might take time to eventually occur, meaning that the detection of this sign would be dependent on the time from ICH onset to imaging. This could not be investigated in the current study because the exact time from onset to imaging was not available, but future studies may shed light on this possibility. Finally, our study required consent from either the patient or a proxy, effectively excluding patients with large, clinically devastating ICH. Overall, this led to our included cohort being less mildly affected than a general ICH cohort.

The implications of this study are that the presence of a blood-fluid level on the CT scan of a patient with ICH should alert clinicians that the patient is likely to be taking an OAC. This is associated with a high risk of hematoma growth and mortality^26^. In such patients, higher-level care, intensive blood pressure control, rapid coagulation assays, and reversal of anticoagulation might be appropriate^27–30^. However, physicians must consider the small chance that, in rare cases, the presence of blood-fluid level may not be associated with OAC use. This is particularly important because blind reversal could lead to adverse events such as thrombosis^30^.

In conclusion, the presence of a blood-fluid level on an acute CT scan is specific for anticoagulant treatment and is not associated with higher mortality at 6 months. Clinicians should be aware that patients with this sign are likely to be taking an OAC, which may help guide the acute management of such patients.

## Supplementary information

Supplementary Table S1.

## Data Availability

Analyses for the CROMIS-2 study are ongoing; once all of these analyses are completed, the CROMIS-2 Steering Committee will consider applications from other researchers for access to anonymised source data.
