# Publisher Correction: Sensitivity and specificity of blood-fluid levels for oral anticoagulant-associated intracerebral haemorrhage

**DOI:** 10.1038/s41598-021-88890-5

**Published:** 2021-04-28

**Authors:** Abeer Almarzouki, Duncan Wilson, Gareth Ambler, Clare Shakeshaft, Hannah Cohen, Tarek Yousry, Rustam Al-Shahi Salman, Gregory Y. H. Lip, Henry Houlden, Martin M. Brown, Keith W. Muir, Hans Rolf Jäger, David J. Werring

**Affiliations:** 1 Physiology Department, Faculty of Medicine, King Abdulaziz University, Jeddah, Saudi Arabia; 2 Department of Brain Repair and Rehabilitation, UCL Stroke Research Centre, UCL Institute of Neurology and the National Hospital for Neurology and Neurosurgery, Russell Square House, 10-12 Russell Square, London, WC1B 5EH UK; 3 Department of Statistical Science, University College London, Gower Street, London, UK; 4 Haemostasis Research Unit, Department of Haematology, University College London, 51 Chenies Mews, London, UK; 5 Lysholm Department of Neuroradiology and the Neuroradiological Academic Unit, Department of Brain Repair and Rehabilitation, UCL Institute of Neurology, Queen Square, London, UK; 6 Centre for Clinical Brain Sciences, School of Clinical Sciences, University of Edinburgh, Edinburgh, UK; 7 Liverpool Centre for Cardiovascular Science, University of Liverpool and Liverpool Heart and Chest Hospital, Liverpool, UK; 8 Aalborg Thrombosis Research Unit, Department of Clinical Medicine, Aalborg University, Aalborg, Denmark; 9 Institute of Neuroscience and Psychology, University of Glasgow, Queen Elizabeth University Hospital, Glasgow, UK; 10 Department of Molecular Neuroscience, UCL Institute of Neurology and the National Hospital for Neurology and Neurosurgery, Queen Square, London, WC1N 3BG UK

Correction to: *Scientific Reports* 10.1038/s41598-020-72504-7, published online 23 September 2020

In the original version of this Article, the author Rustam Al-Shahi Salman was incorrectly indexed. This error has now been corrected.

