# Supplementary material for: Sensitivity and specificity of blood-fluid levels for oral anticoagulant-associated intracerebral haemorrhage
Source: Sci Rep. 2020 Sep 23;10:15529. doi: 10.1038/s41598-020-72504-7 (PMC7511300; doi:10.1038/s41598-020-72504-7)
Supplement: Supplementary file 1 — Supplementary Table S1. [file 41598_2020_72504_MOESM1_ESM.docx]

Sensitivity and specificity of blood-fluid levels for oral anticoagulant-associated intracerebral haemorrhage

Abeer Almarzouki^1^, Duncan Wilson MBChB PhD^2^, Gareth Ambler PhD^3^, Clare Shakeshaft MSc^2^, Hannah Cohen PhD^4^, Tarek Yousry Dr Med Habil FRCR^5^, Rustam Al-Shahi Salman PhD^6^, Gregory Y H Lip FRCP^7,8^, Henry Houlden MRCP^10^, Martin M Brown FRCP^2^, Keith W Muir MD FRCP^9^, Hans Rolf Jäger MD FRCR^5^, David J Werring PhD FRCP^2*^

^1^Physiology Department, Faculty of Medicine, King Abdulaziz University, Jeddah, Saudi Arabia

^2^UCL Stroke Research Centre, Department of Brain Repair and Rehabilitation, UCL Institute of Neurology and the National Hospital for Neurology and Neurosurgery, Russell Square House, 10 - 12 Russell Square, London, UK

^3^Department of Statistical Science, University College London, Gower Street, London, UK

^4^Haemostasis Research Unit, Department of Haematology, University College London, 51 Chenies Mews, London, UK

^5^Lysholm Department of Neuroradiology and the Neuroradiological Academic Unit, Department of Brain Repair and Rehabilitation, UCL Institute of Neurology, Queen Square, London, UK

^6^Centre for Clinical Brain Sciences, School of Clinical Sciences, University of Edinburgh, Edinburgh, UK

^7^Liverpool Centre for Cardiovascular Science, University of Liverpool and Liverpool Heart &

Chest Hospital, Liverpool, United Kingdom

^8^Aalborg Thrombosis Research Unit, Department of Clinical Medicine, Aalborg University, Aalborg, Denmark

^9^Institute of Neuroscience & Psychology, University of Glasgow, Queen Elizabeth University Hospital, Glasgow, UK

^10^Department of Molecular Neuroscience, UCL Institute of Neurology and the National Hospital for Neurology and Neurosurgery, Queen Square, London WC1N 3BG

*Corresponding author: Professor David J Werring [d.werring@ucl.ac.uk](mailto:d.werring@ucl.ac.uk)

UCL Stroke Research Centre, Department of Brain Repair and Rehabilitation, UCL Institute of Neurology, Russell Square House, 10 - 12 Russell Square, London WC1B 5EH, UK.

Tel.: +44 (0)20 3108 7493; Fax: +44 (0)20 7833 8613; Email: d.werring@ucl.ac.uk

**Keywords**

Blood-fluid level, fluid-blood level, fluid-fluid level, haematocrit, sedimentation level, blood-fluid interface, fluid-contrast interfaces, intracerebral haemorrhage (ICH), oral anticoagulant-associated ICH, oral anticoagulants, computed tomography (CT).

Supplementary material

*Table 1 - Contingency table showing the relationship between blood-fluid level and anticoagulation in the study sample.*

|  | | **Anticoagulation** | |
| --- | --- | --- | --- |
|  |  | **Yes** | **No** |
| **Blood-fluid level** | **Yes** | 15 | 3 |
|  | **No** | 345 | 492 |
